# Weakly‐Coordinating Non‐Fluorinated Diluents for Local High‐Concentration Electrolytes in Advanced Lithium Metal Batteries

**DOI:** 10.1002/adma.73388

**Published:** 2026-05-20

**Authors:** Yin Cui, Xinrui Li, Nan Jiang, Xinyan Huang, E. Lora da Silva, Ruo Zhao, Tao Yang, Qingxia Liu, Xidong Lin

**Affiliations:** ^1^ Future Technology School Shenzhen Technology University Shenzhen China; ^2^ Department of Building Environment and Energy Engineering The Hong Kong Polytechnic University Kowloon Hong Kong China; ^3^ IFIMUP Institute of Physics for Advanced Materials Nanotechnology and Photonics Department of Physics and Astronomy Faculty of Sciences University of Porto Porto Portugal; ^4^ Institute for Advanced Study Shenzhen University Shenzhen China

**Keywords:** fluorine, liquid electrolytes, lithium metal batteries, local high‐concentration electrolytes, non‐fluorinated diluents

## Abstract

Local high‐concentration electrolytes (LHCEs) resolve the contradiction between ion transport efficiency and interface stability of traditional electrolytes in lithium metal batteries (LMBs) through the innovative design of macroscopically low viscosity and locally high concentration. Nevertheless, fluorinated diluents widely used in current LHCEs possess inherent defects, including high environmental toxicity, poor biodegradability, and high synthesis costs, which severely restrict the sustainable and large‐scale application of LMBs. Recently, non‐fluorinated diluents have attracted growing attention as viable alternatives, yet their roles remain insufficiently understood and often oversimplified as inert viscosity modifiers. In this perspective, we systematically classify non‐fluorinated diluents into chlorinated and halogen‐free systems, and further correlate their molecular structures with solvation behavior, interfacial chemistry, and electrochemical performance. By critically analyzing recent advances, we reveal that non‐fluorinated diluents are not intrinsically benign or universally inert; instead, their effectiveness arises from a delicate balance between weak interactions, transport regulation, and interphase formation. Finally, we outline key challenges and future directions, including parameter‐guided molecular design, multi‐component electrolyte engineering, advanced in situ characterization, and sustainability‐driven evaluation. This perspective aims to provide comprehensive theoretical support for the structural optimization of non‐fluorinated diluents and the innovation of electrolyte formulations, thus promoting the technological progress and industrialization of LMBs.

## Graphical abstract

This perspective critically evaluates non‐fluorinated diluents in local high‐concentration electrolytes, categorizing their solvation behavior, interfacial mechanisms, and sustainability potential to guide future electrolyte design for lithium metal batteries.

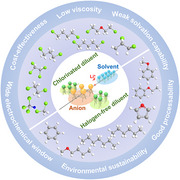

## 1 Introduction

With the rapid development of electric vehicles, portable electronic devices, and large‐scale energy storage stations, the demand for high‐energy‐density batteries is becoming increasingly urgent [1, 2]. Lithium (Li) metal anode, with ultrahigh theoretical specific capacity (3860 mAh g^−1^) and the lowest redox potential (−3.04 V vs. standard hydrogen electrode), is regarded as a pivotal technical pathway to attain the goal of high energy density [3, 4, 5]. However, Li metal anodes suffer from Li dendrite growth, volume expansion, and liquid electrolytes (LEs) decomposition during the cycling process, leading to low Coulombic efficiency (CE), short cycle life, and even safety hazards, which severely impede the practical application of Li metal batteries (LMBs) [6, 7, 8, 9]. Furthermore, as the “blood” of LMBs, the LEs directly determine batteries’ electrochemical behavior and safety performance. The development of high‐performance LEs compatible with LMBs is thus becoming a critical bottleneck in current research [10, 11].

**FIGURE 1:**
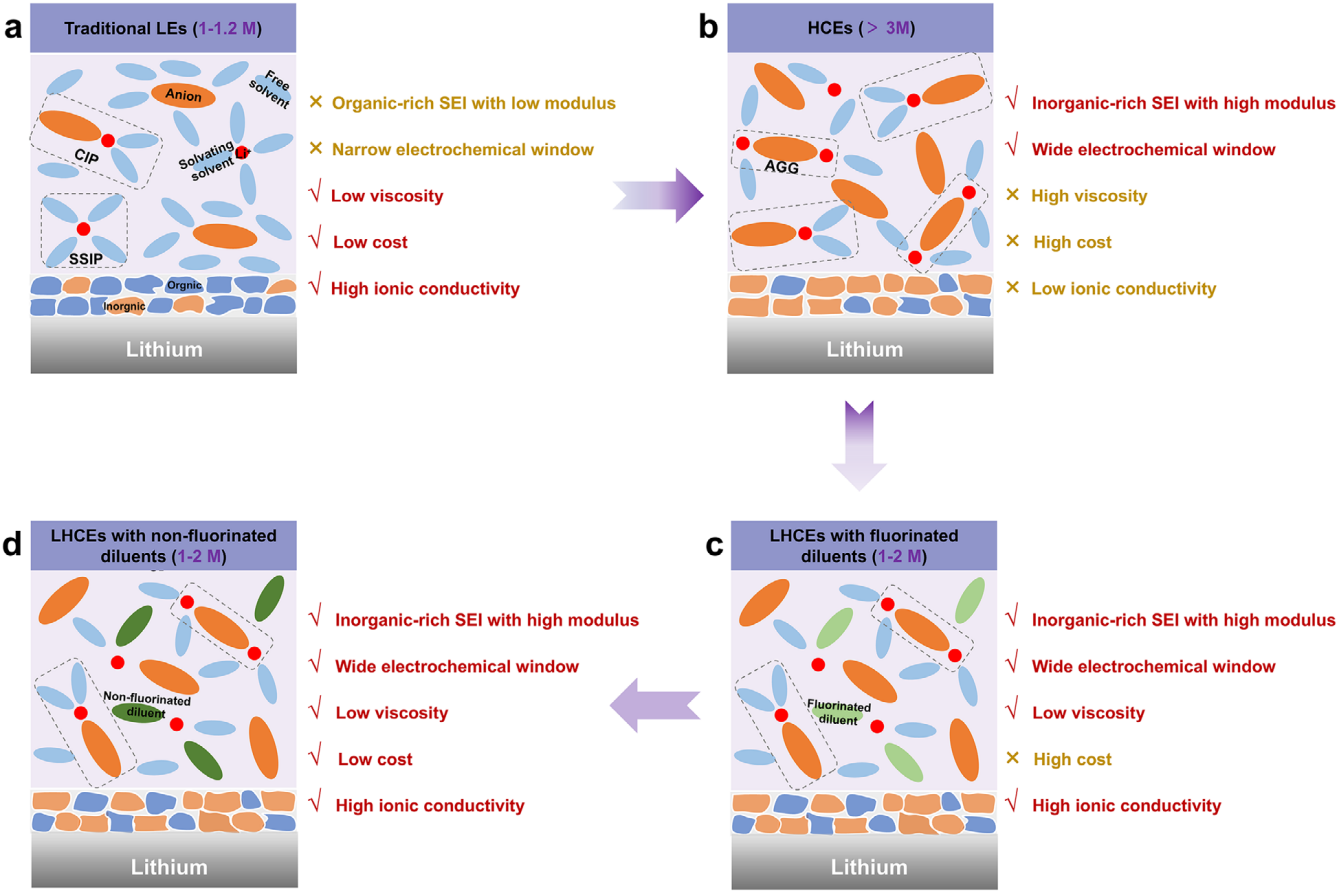
Solvation structures and typical characteristics of (a) traditional LEs, (b) HCEs, (c) LHCEs with fluorinated diluents, and (d) LHCEs with non‐fluorinated diluents.

The macroscopic properties (e.g., ionic conductivity and interface stability) and electrochemical properties of the LEs are closely related to their microscopic solvation structures [12]. These structures are determined by the competitive intermolecular interactions among the various components within the LEs. Generally, the species in the traditional LEs include Li^+^ cations, corresponding salt anions, and different dipolar solvent molecules. Of note, there are inherent contradictions in the concentration design of traditional LEs. Usually, traditional LEs possess low viscosity and rapid ion transport, but their solvation structure, characterized by solvent‐separated ion pairs (SSIPs), contains abundant free solvent molecules, which are prone to decomposition on the Li metal anode surface, disrupting the interface stability (Figure 1a) [6]. High‐concentration electrolytes (HCEs) can form a “Li^+^‐solvent‐anion” cooperative solvation structure, inhibiting solvent decomposition and promoting the formation of a robust inorganic‐rich solid electrolyte interphase (SEI) layer (Figure 1b) [13, 14]. Nevertheless, their high viscosity results in low ion transport efficiency and poor rate performance [15, 16]. To balance “high‐concentration solvation” and “low‐viscosity processability”, localized high‐concentration electrolytes (LHCEs) by introducing diluents (including fluorinated and non‐fluorinated diluents) into the HCEs have emerged as a promising solution (Figure 1c,d) [17, 18, 19, 20]. These weakly polar diluents exhibit extremely limited salt solubility and minimal interaction with Li^+^, thereby maintaining the high content of contact ion pairs (CIPs) and aggregates (AGGs) [21, 22, 23]. Consequently, LHCEs retain the interface stability characteristics of HCEs and the low‐viscosity advantage of traditional LEs, effectively achieving the synergistic optimization of ion transport efficiency and interface compatibility.

Currently, most diluents used in LHCEs are fluorinated compounds, such as fluorinated ethers (e.g., 1,1,2,2‐tetrafluoro‐1‐methoxyethane, 1,1,2,2‐tetrafluoroethyl‐2,2,3,3‐tetrafluoropropyl ether, 1,1,2,2,3,3,4,4‐octafluoro‐5‐(1,1,2,2‐tetrafluoroethoxy)pentane, and bis(2,2,2‐trifluoroethyl) ether) [24, 25, 26, 27], fluoroaromatic hydrocarbons (e.g., fluorobenzene, 1,2‐difluorobenzene, and m‐fluorotoluene) [28, 29, 30], and fluorinated esters (e.g., difluoroethylene carbonate and 3,3,3‐trifluoropropionate) [31, 32]. Among them, fluorinated ethers can enhance the anion‐cation interactions, thereby promoting the formation of AGGs. Owing to the fluorine (F) atoms with strong electron‐withdrawing ability, fluorinated ethers typically exhibit high oxidative stability, which is beneficial for their application in high‐voltage LMBs. Compared with fluorinated ethers, fluoroaromatic hydrocarbons have the advantages of low cost and low density, and when used as diluents, they help to construct a homogeneous and robust SEI layer. In contrast to ether‐based compounds, fluorinated esters possess higher boiling points, enabling their use at high temperatures, and are compatible with high‐voltage cathodes.

Although fluorinated diluents can effectively reduce the electrolyte viscosity and improve the oxidative stability, there are undeniable drawbacks. First, fluorinated diluents, which often exhibit environmental persistence similar to that of per‐ and polyfluoroalkyl substances (PFAS) due to their multiple C─F bonds, are prone to accumulation in the environment, causing long‐term adverse impacts on the ecosystem [33]. Second, certain fluorinated solvents exhibit considerable biological toxicity, potentially irritating the human respiratory system and skin, while also generating toxic intermediates during the synthesis process [34]. Third, the introduction of fluorinated groups increases the complexity of the synthesis process, leading to significant challenges in large‐scale production and persistently high cost [35]. Finally, fluorinated diluents can accelerate the decomposition of the Li metal anode, resulting in the electrolyte desiccation and rendering batteries highly susceptible to failure [36, 37]. These drawbacks make fluorinated diluents unable to meet the requirements of green and sustainable industrial development. In particular, in practical and regulatory contexts, many fluorinated diluents used in battery electrolytes are either classified as PFAS or exhibit PFAS‐like persistence, thereby motivating the search for non‐fluorinated alternatives. Non‐fluorinated diluents provide PFAS‐free alternatives to fluorinated solvents, particularly as they mitigate concerns associated with long‐term environmental persistence. Consequently, the development of non‐fluorinated compounds has become an inevitable trend in the technological advancement of electrolytes.

**FIGURE 2:**
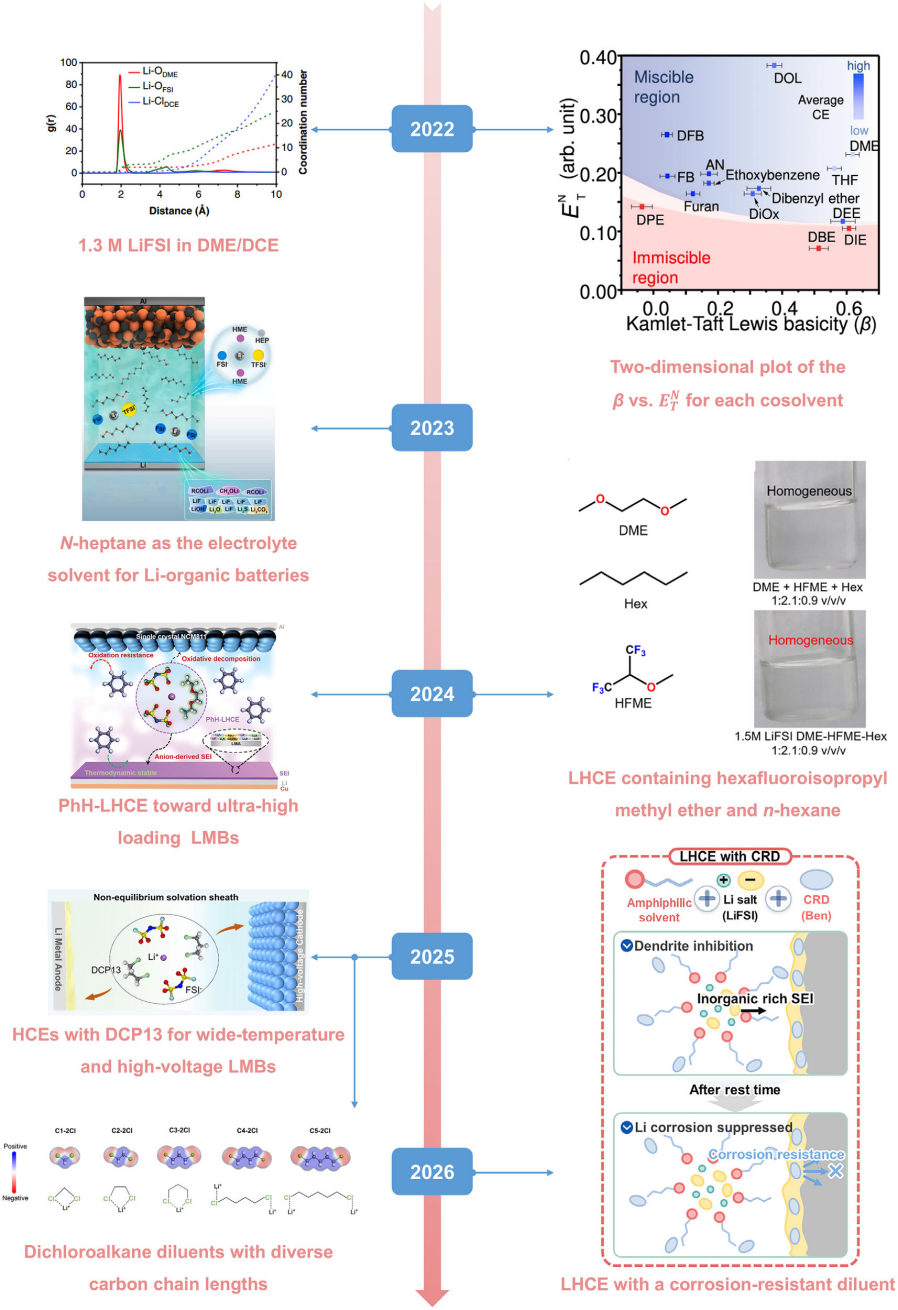
The evolution timeline of LHCEs with non‐fluorinated diluents. Reproduced with permission [38]. Copyright 2022, Nature Publishing Group. Reproduced with permission [39]. Copyright 2022, Nature Publishing Group. Reproduced with permission [40]. Copyright 2023, Elsevier B.V. All rights reserved. Reproduced with permission [41]. Copyright 2024, Wiley‐VCH GmbH. Reproduced with permission [42]. Copyright 2024, The Royal Society of Chemistry. Reproduced with permission [43]. Copyright 2024, Wiley‐VCH GmbH. Reproduced with permission [44]. Copyright 2025, Nature Research. Reproduced with permission [45]. Copyright 2026, Wiley‐VCH GmbH.

Accordingly, recent studies have increasingly shifted attention toward non‐fluorinated diluents (e.g., ethoxybenzene, furan, anisole (AN), and benzene) as functional components in LHCEs for LMBs, and have achieved breakthroughs (Figure 2) [38, 39, 40, 41, 42, 43, 44, 45]. While some non‐fluorinated diluents primarily function as viscosity modifiers, an increasing number of studies demonstrate that certain diluents can exert subtle yet consequential influences on Li^+^ solvation environments and interfacial reaction pathways through weak, dynamic, and statistically minor interactions. This highlights the chemical diversity of non‐fluorinated diluents beyond simple viscosity modifiers.

In this perspective, we build upon recent advances of non‐fluorinated diluents in LHCEs to reorganize them into chemically meaningful categories, and perform explicit structure‐performance comparisons within each class. We further provide a deeper understanding of the role of non‐fluorinated diluents in regulating solvation structures and interfacial chemistry, thereby underpinning the electrochemical behavior of LMBs. Finally, we conclude with critical insights and an outlook on the development of LHCEs with non‐fluorinated diluents, with an emphasis on balancing electrochemical performance, safety considerations, and practical applicability.

## 2 Design Principles of LHCEs with Non‐Fluorinated Diluents

LHCEs composed of Li salts, main solvents, and diluents integrate the excellent interfacial stability of high‐concentration electrolytes with the advantages of low viscosity and high wettability [46, 47]. However, the conventional selection of LHCEs components has largely relied on empirical trial‐and‐error approaches, and the matching mechanism among main solvents, Li salts, and diluents has long lacked a unified theoretical framework. Recently, advances in solvation thermodynamics have overcome the traditional limitation of describing solvation ability solely in terms of solvation enthalpy [48]. Meanwhile, a quantitative theory based on normalized cation‐solvent and anion‐solvent affinities (*α*_s_ and *β*_s_) has provided a general principle for the precise design and efficient screening of the three components in LHCEs [49].

### 2.1 Li Salts

Li salts are the sole source of Li^+^ in LHCEs, and their structure and performance directly determine the ion transport efficiency, electrochemical stability, and interface reaction characteristics of the electrolyte. Currently, the commonly used Li salts in LHCEs are mainly fluorinated anion salts, including lithium hexafluorophosphate (LiPF_6_), lithium bis(fluorosulfonyl)imide (LiFSI), and lithium bis(trifluoromethane)sulfonimide (LiTFSI). Meanwhile, there are also explorations of using Li salts such as Li difluorobis(oxalato) phosphate as an additive in LHCEs [50].

From the perspective of structural characteristics, the anion size, electronegativity, and stability of the Li salts are the key influencing factors. LiPF_6_ is the dominant choice for commercial electrolytes due to its high ion dissociation efficiency and low cost, but it exhibits a relatively low thermal decomposition onset temperature, despite its comparatively lower total heat release during thermal runaway. Benefiting from larger anion sizes and stronger electron delocalization, LiFSI and LiTFSI show higher thermal stability and ionic conductivity. Additionally, their decomposition products contain LiF, which can optimize the composition of the SEI layer. However, LiFSI and LiTFSI pose a corrosion risk to aluminum current collectors, requiring the use of additives or optimization of electrolyte components [51]. In the LHCEs system, the Li salt concentration is usually up to 1.0‐2.0 mol L^−1^, which is lower than that of HCEs (>3 mol L^−1^) and higher than that of traditional electrolytes (1.0–1.2 mol L^−1^) [47]. A large proportion of the anions coordinate with Li^+^ to form CIPs and AGGs. The non‐fluorinated diluents need to meet the core requirement of “not dissolving Li salts” to avoid disrupting the solvation equilibrium, so the dissolution of the Li salts mainly depends on the polarizing ability and coordination of the main organic solvents.

### 2.2 Main Organic Solvents

**FIGURE 3:**
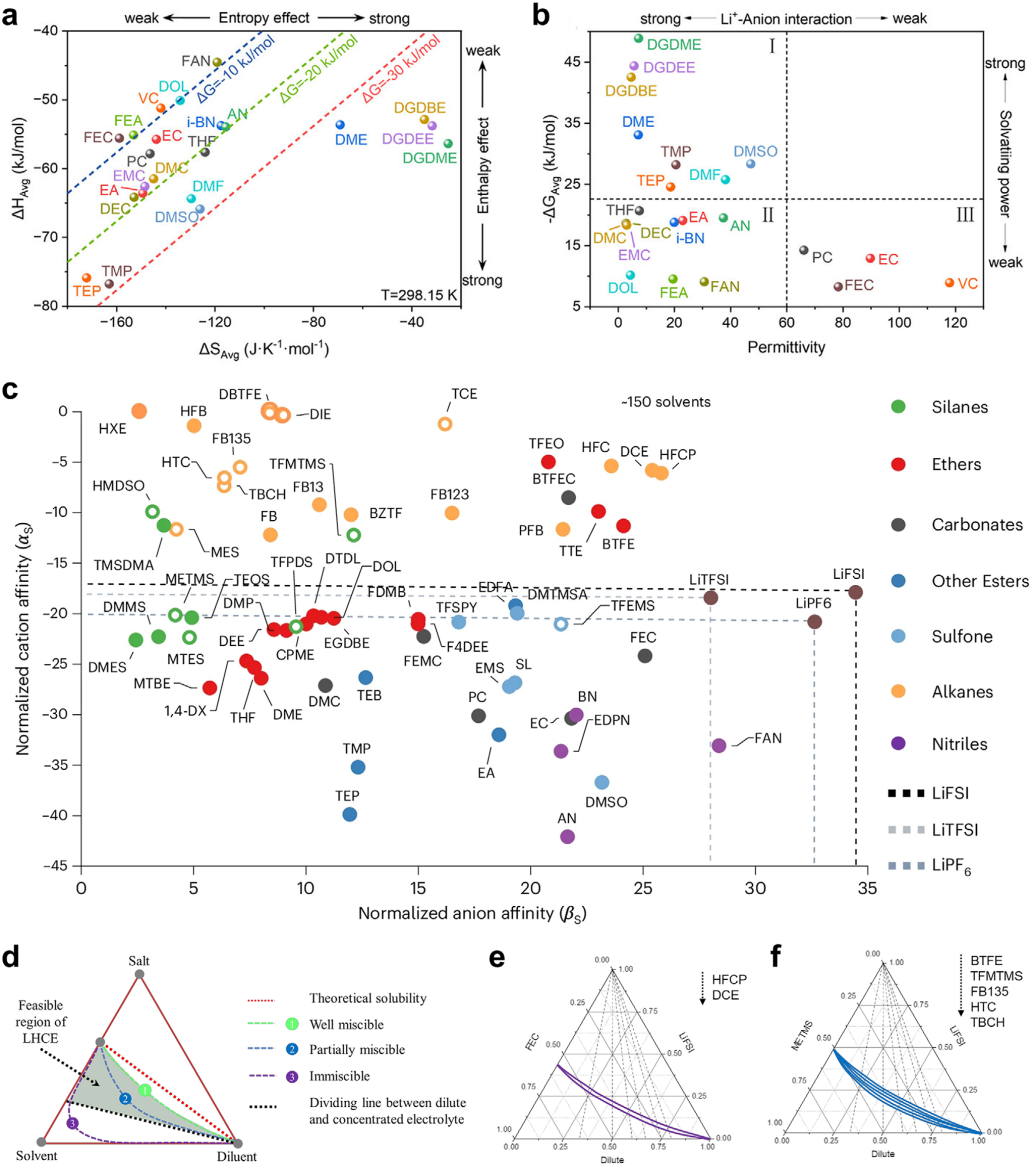
(a) The ∆*H_Avg_*‐∆*S_Avg_* diagram of a series of common solvents. (b) The ∆*G_Avg_* and permittivity of a series of common solvents. Panels a,b) Reproduced with permission [48]. Copyright 2025, Wiley‐VCH GmbH. (c) The normalized ion‐solvent affinity database for various Solvents. (d) LHCEs design principles and the corresponding ternary phase diagram of Li salt, solvent, and diluent. The ternary phase diagram is divided into three regions: the solution region (green area), the phase segregation region (red area), and the salt precipitation region (grey area). LHCEs dissolution experiment based on (e) FEC and (f) METMS. The dissolution curves in the figure are divided according to the diluent indicated by the arrows. Panels c–f) Reproduced with permission [49]. Copyright 2025, Nature Publishing Group.

As the core component of LHCEs responsible for dissolving Li salts and constructing Li^+^ solvation sheaths, main solvents are required to exhibit high dielectric constants and low viscosities [52, 53]. In addition, the main solvents should have wide electrochemical stability windows and excellent chemical stability to reduce the occurrence of side reactions. Notably, systematic analyses based on solvation thermodynamics demonstrate that conventional approaches relying only on solvation enthalpy, such as donor number (DN) or binding energy, exhibit significant limitations in evaluating solvation capability [48]. The two‐dimensional map of average solvation enthalpy (∆*H_Avg_*) and entropy (∆*S_Avg_*) established in Figure 3a clearly shows that multidentate ether solvents, including 1,2‐dimethoxyethane (DME) and diethylene glycol dimethyl ether (DGDME), despite relatively weak Li^+^‐O interactions, occupy a thermodynamically favorable region due to their exceptionally high solvation entropy. As a result, they exhibit solvation abilities that surpass those of conventional solvents. These findings confirm the decisive role of solvation entropy in chelating solvents. Building on this understanding, Figure 3b further presents a general electrolyte solvent design database using average solvation free energy (∆*G_Avg_*) and dielectric constant as key descriptors. Based on differences in solvation ability and dielectric constant, solvents can be classified into three categories: strongly solvating and low‐dielectric ether systems, weakly solvating and low‐dielectric systems, and weakly solvating and high‐dielectric conventional carbonate systems. This classification provides an intuitive representation of the competitive behavior of solvents within the solvation sheath and the resulting solvation structures. It also establishes quantitative design principles for constructing solvent‐dominated, anion‐dominated, or SSIP configurations.

Furthermore, according to the normalized ion‐solvent affinity database for various solvents, the selection of main solvents should be guided by a moderate yet effective ion affinity (Figure 3c) [49]. Specifically, main solvents must possess sufficient coordination ability toward Li^+^, with *α*_s_ slightly lower than or close to the affinity of the salt for the anion (*α*_salt_), to ensure effective dissociation of Li salts. At the same time, their affinity toward anions should remain relatively weak to suppress excessive solvation of anions and promote their participation in the primary solvation sheath. This characteristic enables the formation of CIPs or AGGs, even at low or moderate concentrations, resembling those found in HCEs, thereby facilitating the formation of stable SEI enriched in inorganic components such as LiF. In other words, ideal main solvents should lie within a window that balances salt solubility with controlled anion solvation, achieving a trade‐off between dissolution capability and structural regulation.

Currently, the mainstream solvents are mainly divided into two categories: carbonate esters and ethers. These two types of solvents are often used in a combined form to balance the solubility and viscosity. Carbonate ester‐based main solvents include cyclic carbonates (e.g., ethylene carbonate and propylene carbonate) and linear carbonates (e.g., dimethyl carbonate and ethyl methyl carbonate). Among them, cyclic carbonates, with their high dielectric constants and strong coordination abilities, are the core components for dissolving Li salts, but they show relatively high viscosities. Linear carbonates exhibit lower viscosities and are mainly used to regulate the fluidity of the electrolyte, and when combined with cyclic carbonates, they can achieve the synergy of high solubility and low viscosity. Ether‐based main solvents (e.g., DME and 1,3‐dioxolane) have lower dielectric constants than those of carbonate ester‐based solvents, but they exhibit strong Lewis basicity, outstanding coordination ability with Li^+^, and extremely low viscosity. They are particularly suitable for Li‐sulfur and Li‐air battery systems. The solvation structures formed by ether‐based main solvents and Li salts are more conducive to the participation of anions in coordination, which is beneficial for improving the Li^+^ transference number. Regardless of the type, the main solvents need to have good miscibility and stability with the diluent. Their solubility parameters should match the diluents to avoid phase separation of LHCEs during storage or cycling.

### 2.3 Non‐Fluorinated Diluents

Diluents play a critical role in enabling the low viscosity and high ionic conductivity of LHCEs. Diluents are generally miscible with main solvents but are immiscible with or exhibit poor solubility in Li salts [54]. Their role is not that of inert solvents, but rather critical components that participate in modulating the solvation structure [55]. The quantitative design principles for diluents consist of two criteria [49]. First, *α*_s_ (diluents) > *α*_salt_, ensuring that the diluents do not dissolve Li salts. Second, *β*_s_ (diluents) > *β*_s_ (main solvent), meaning that diluents exhibit stronger affinity toward anions than the main solvents do, thereby preventing the collapse of the solvation structure or phase separation upon dilution. Ternary phase diagram analyses provide direct validation of these criteria (Figure 3d). As *β*_s_ of the main solvents increases, the requirement for matching *β*_s_ of the diluents becomes more stringent. For example, high‐*β*_s_ systems such as fluoroethylene carbonate (FEC) (Figure 3e) can only be paired with diluents possessing similarly high *β*_s_ values, such as 1,1,2,2,3,3,4‐heptafluorocyclopentane (HFCP) or 1,2‐dichloroethane (DCE), whereas low‐*β*_s_ systems such as methoxy(trimethyl)silane (METMS) (Figure 3f) exhibit a broader compatibility window for diluents.

The diluents can be classified into fluorinated diluents and non‐fluorinated diluents based on whether they contain F element. On the one hand, the non‐fluorinated diluents must meet certain basic requirements. For instance, the non‐fluorinated diluent must have a low dielectric constant and maintain a limited solubility for Li salts, thereby preserving the local solvation environment of the inner solvation shell in HCEs. They should also have low viscosity, which can effectively reduce the macroscopic viscosity of LHCEs and enhance the transfer efficiency of Li^+^. Furthermore, they need to possess excellent chemical stability, avoiding direct reactions with Li metal, and should promote the formation of a dense SEI layer to avoid a rough interface caused by the decomposition of the diluents. Meanwhile, they also need to meet safety requirements such as high flash point and low volatility, so as to reduce the risk of battery thermal runaway.

On the other hand, according to relevant reports, parameters such as the Kamlet‐Taft Lewis basicity (*β*), normalized molar electron transition energy ($E_{T}^{N}$), average coordination number (CN), binding energy, DN, and maximum electrostatic potential have been proposed as key indicators for evaluating the non‐fluorinated diluents. For example, to assess the solvation ability of a solvent, it is necessary to determine the stabilization energy of Li^+^ in a specific solvation structure, that is, the energy difference between the solvated state and the unsolvated state of the Li^+^. Lim et al., selected two dyes that could form solvation structures with the target solvent to simulate this definition [39]. Specifically, the dye containing a primary amine group (─NH_2_) can form hydrogen bonds with solvent molecules, while the dye containing a tertiary amine group (–N(CH_2_CH_3_)_2_) lacks this effect. Given that the hydrogen atom in the –NH_2_ experiences a solvation environment equivalent to that of Li^+^, this similarity can be used to estimate the stabilization energy of Li^+^ in the target solvent. Since the strong interaction between the electrons of the solvent molecules and the Li^+^ is similar to the Lewis acid‐base interaction, the solvation ability of the solvent is characterized by the *β* parameter [56]. Of note, the research has found that cosolvents with a lower *β* value tend to weaken competitive Li^+^‐solvent coordination, thereby increasing the proportion of CIP and AGG relative to SSIP ((CIP + AGG)/SSIP) [57]. Such a shift toward a higher (CIP + AGG)/SSIP ratio effectively enhances anion involvement in the Li^+^ solvation environment, which is considered beneficial for the formation of LHCEs.

Although traditional parameters such as dielectric constant and dipole moment are based on the “like dissolves like” principle to guide the molecular miscibility, they still have limitations in precise measurement. The $E_{T}^{N}$ is a solvatochromic parameter proposed by Reichardt, which is used to characterize solvent miscibility [58]. It can directly measure the stabilization energy of solvents in high‐polarity electrolytes, which arises from the intermolecular interactions between components. This energy corresponds to the difference between the energy of the solvent in its isolated state and that in the electrolyte environment. $E_{T}^{N}$ can more accurately reflect the solubility of solvents by characterizing the intermolecular forces between the target solvent and polar substances representing the highly polar environment of the electrolyte, which is different from the traditional parameters of pure substances. It has been proved that the non‐fluorinated diluent should have a relatively high $E_{T}^{N}$ value (>0.11) to be miscible with the polar electrolyte solvent [39].

In addition, the CN and binding energy are also key quantitative parameters for describing the interaction between non‐fluorinated diluents and Li^+^, as well as the characteristics of the solvation structures. The CN is obtained by integrating the radial distribution function (RDF) of Li^+^ and characteristic atoms in the non‐fluorinated diluent molecules, which reflects the degree of participation of the non‐fluorinated diluents in Li^+^ solvation [59, 60, 61]. If the average CN of the non‐fluorinated diluents with Li^+^ is low, it indicates extremely weak interaction, and the diluents cannot enter the inner solvation shell of Li^+^. Instead, the non‐fluorinated diluents only act as inert diluents to disperse the CIPs and AGGs formed by the main solvent and Li salt, thereby maintaining the structural advantage of “local high concentration” in LHCEs. The binding energy between the non‐fluorinated diluents and Li^+^ is calculated via density functional theory (DFT), directly characterizing the solvation ability of the non‐fluorinated diluents [62, 63]. When the absolute value of the binding energy is low, it demonstrates that the coordination of the non‐fluorinated diluents with Li^+^ is weak, and that these diluents cannot effectively compete for the coordination sites of Li^+^ with the main solvent. This not only avoids the disruption of the local solvation structure by the non‐fluorinated diluents, but also assists in regulating the viscosity of the electrolyte through weak interactions. The combined analysis of these two parameters provides a key theoretical basis for screening “miscible yet salt‐insoluble” non‐fluorinated diluents suitable for LHCEs, and also serves as the core criterion for verifying whether such diluents meet the functional requirements of LHCEs. Importantly, the computational parameters cited in this work are derived from the original literature. These values are intended to convey qualitative trends and mechanistic insights reported in each study, rather than to serve as absolute benchmarks for direct quantitative comparison across systems.

Noteworthily, to bridge the design principles and practical behaviors of non‐fluorinated diluents in LHCEs, it is essential to consider their solvation characteristics under both equilibrium and non‐equilibrium conditions. Under non‐equilibrium conditions, such as in the presence of an external electric field, certain diluents (e.g., 1,3‐dichloropropane (DCP13)) can participate in Li^+^ coordination, forming Li^+^‐diluent complexes that influence ion transport and interfacial chemistry [43]. This behavior arises from the intrinsic but weak binding affinity between Li^+^ and diluent molecules. In contrast, under equilibrium conditions, most non‐fluorinated diluents (e.g., AN, ethoxybenzene, furan, and *n*‐heptane) exhibit an average CN with Li^+^ that is approximately zero, indicating negligible participation in the primary solvation sheath and a genuinely non‐solvating character [39]. These diluents are statistically excluded from the first solvation shell and primarily act as inert media that preserve the anion‐rich solvation structure dominated by CIPs and AGGs. Meanwhile, a limited subset of diluents (e.g., DCP13, DCE, and 1,4‐dichlorobutane) shows weak but non‐negligible coordination with Li^+^, where occasional involvement in the first solvation shell may occur without dominating the overall solvation structure [44, 64].

Therefore, the “non‐solvating” nature of non‐fluorinated diluents in LHCEs should be understood as a statistical descriptor rather than an absolute absence of interaction. The coexistence of equilibrium non‐solvation and condition‐dependent coordination reflects the intrinsic solvation characteristics of non‐fluorinated diluents and provides a unified framework for interpreting their roles in practical electrolyte systems. Furthermore, certain components may facilitate the formation of anion‐rich solvation structures; however, if they consistently coordinate with Li^+^ and enter the primary solvation sheath under both equilibrium and non‐equilibrium conditions, they should be classified as co‐solvents rather than true diluents [65].

## 3 Non‐Fluorinated Diluents and Their Applications

Based on whether halogen atoms (except for F) are present in the molecular structure, non‐fluorinated diluents can be classified into two main categories: chlorinated diluents [38, 43, 64, 66, 67] and halogen‐free diluents [39, 40, 41, 42, 45, 68]. Table 1 summarizes the chemical structures and basic parameters of the non‐fluorinated diluents employed in previously reported LHCEs. On the one hand, chlorinated diluents regulate the molecular polarity and interfacial interaction characteristics via introducing chlorine atoms, and demonstrate unique advantages in optimizing the stability of electrolytes and the quality of the electrode/electrolyte interface. They have emerged as a prominent research direction compatible with LHCEs. On the other hand, halogen‐free diluents are characterized by the absence of any halogen atoms and possess merits such as environmental friendliness and low synthesis cost. They also represent the core direction for the sustainable development of electrolytes.

**TABLE 1:** Various non‐fluorinated diluents in previously published literatures.

Category		Table	Diluents	Molecular Structure	Ρ [g cm^−3^]	Permittivity	Flash Point [°C]	Boiling Point [°C]
chlorinated diluents	chlorinated alkanes	1	dichloromethane	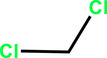	1.33	9.10	−	39
		2	chloroform‐d	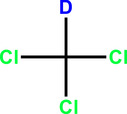	1.50	4.72	62	61
		3	1,2‐dichloroethane	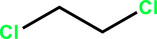	1.26	10.70	16	84
		4	1,1,2,2‐tetrachloroethane	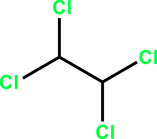	1.59	8.42	144	146
		5	1,2‐dichloropropane	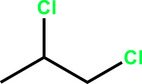	1.16	9.00	16	96
		6	1,3‐dichloropropane	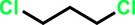	1.19	9.50	16	121
		7	1,4‐dichlorobutane	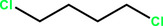	1.16	9.65	40	154
		8	1,5‐dichloropentane	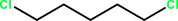	1.11	9.12	27	141
	chlorinated ether	9	2,2‐dichlorodiethyl ether	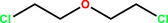	1.22	20.79	55	179
halogen‐free diluents	π‐conjugated aromatics	10	benzene	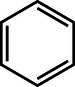	0.87	2.30	−11	80
		11	anisole	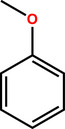	0.99	4.30	52	156
		12	ethoxybenzene	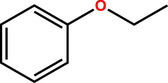	0.97	4.20	63	172
		13	furan	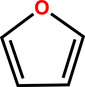	0.94	3.00	−35	32
	alkanes	14	*n*‐hexane	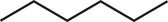	0.66	2.00	−22	69
		15	*n*‐heptane	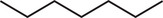	0.68	1.90	−1	98

### 3.1 Chlorinated Diluents

Apart from fluorides, chlorides have demonstrated great potential in enhancing the performance of electrolytes [43, 44, 64, 67, 69]. Although chlorinated diluents are typically designed not to participate in Li^+^ solvation, certain chlorinated diluents exhibit weak but non‐negligible interactions with Li^+^ under equilibrium conditions. In contrast, they can participate in coordination under non‐equilibrium environments.

**FIGURE 4:**
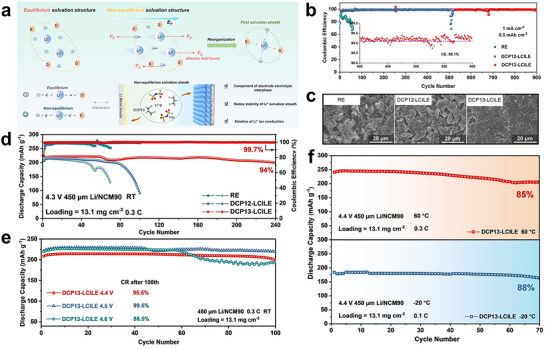
(a) Schematic diagram of the transition from equilibrium solvation structure to non‐equilibrium solvation structure under an external electric field. (b) CE of Li/Cu cells with reference electrolyte (RE, 1 M LiPF_6_ in ethylene carbonate/diethyl carbonate (1:1 by volume)), 1,2‐dichloropropane (DCP12) ‐LCILE and DCP13‐LCILE at 1 mA cm^−2^ and 0.5 mAh cm^−2^. (c) Top‐view scanning electron microscopy (SEM) morphologies of the surface of Li plated on Cu foils in RE, DCP12‐LCILE, and DCP13‐LCILE. (d) Cycling performance of Li/NCM90 cells with a charge cutoff voltage of 4.3 V at 0.3 C with RE, DCP12‐LCILE, and DCP13‐LCILE. (e) Cycling performance of Li|DCP13‐LCILE|NCM90 cells at 4.4, 4.5, and 4.6 V. (f) The cycling performance of Li|DCP13‐LCILE|NCM90 cells at 4.4 V under 60 °C and −20 °C. Reproduced with permission [43]. Copyright 2024, Wiley‐VCH GmbH.

Wu et al., developed a wide‐temperature locally concentrated ionic liquid electrolyte (DCP13‐LCILE) using chloroalkane solvent DCP13 as the diluent (Figure 4a) [43]. Contrary to the solvation structure of conventional LHCEs, the team found that DCP13 would participate in the solvation structure of Li^+^ under an electric field, thereby affecting battery performance. In this non‐equilibrium solvation structure, the DCP13 diluent molecules exhibit high affinity for Li^+^, reducing the content of free diluent molecules and thus enhancing the oxidative stability of the electrolyte. The Li|DCP13‐LCILE|Cu cell shows stable cycling performance at 1 mA cm^−2^ and 0.5 mAh cm^−2^ for more than 900 cycles, achieving a high average CE of 99.1% (Figure 4b). Based on the experimental observations, the coordination between DCP13 and Li^+^ in the non‐equilibrium solvation structure is associated with enhanced transport kinetics and the formation of a dense and smooth Li metal deposition morphology (Figure 4c). Therefore, the cell assembled with a high‐nickel cathode (LiNi_0.9_Co_0.05_Mn_0.05_O_2_, NCM90), DCP13‐LCILE, and Li metal anode achieves a high‐capacity retention of 94% after 240 cycles at 4.3 V (Figure 4d). Importantly, the Li|DCP13‐LCILE|NCM90 cells demonstrate excellent stability at high cut‐off voltages of 4.4 to 4.6 V and a wide temperature range of −20 °C to 60 °C (Figure 4e,f). This work presents a dynamic perspective of the non‐equilibrium solvation structure of electrolytes under an applied electric field, provides new insights into the role of diluents in LCILEs, and offers new inspirations for the design of high‐performance electrolytes for LMBs.

**FIGURE 5:**
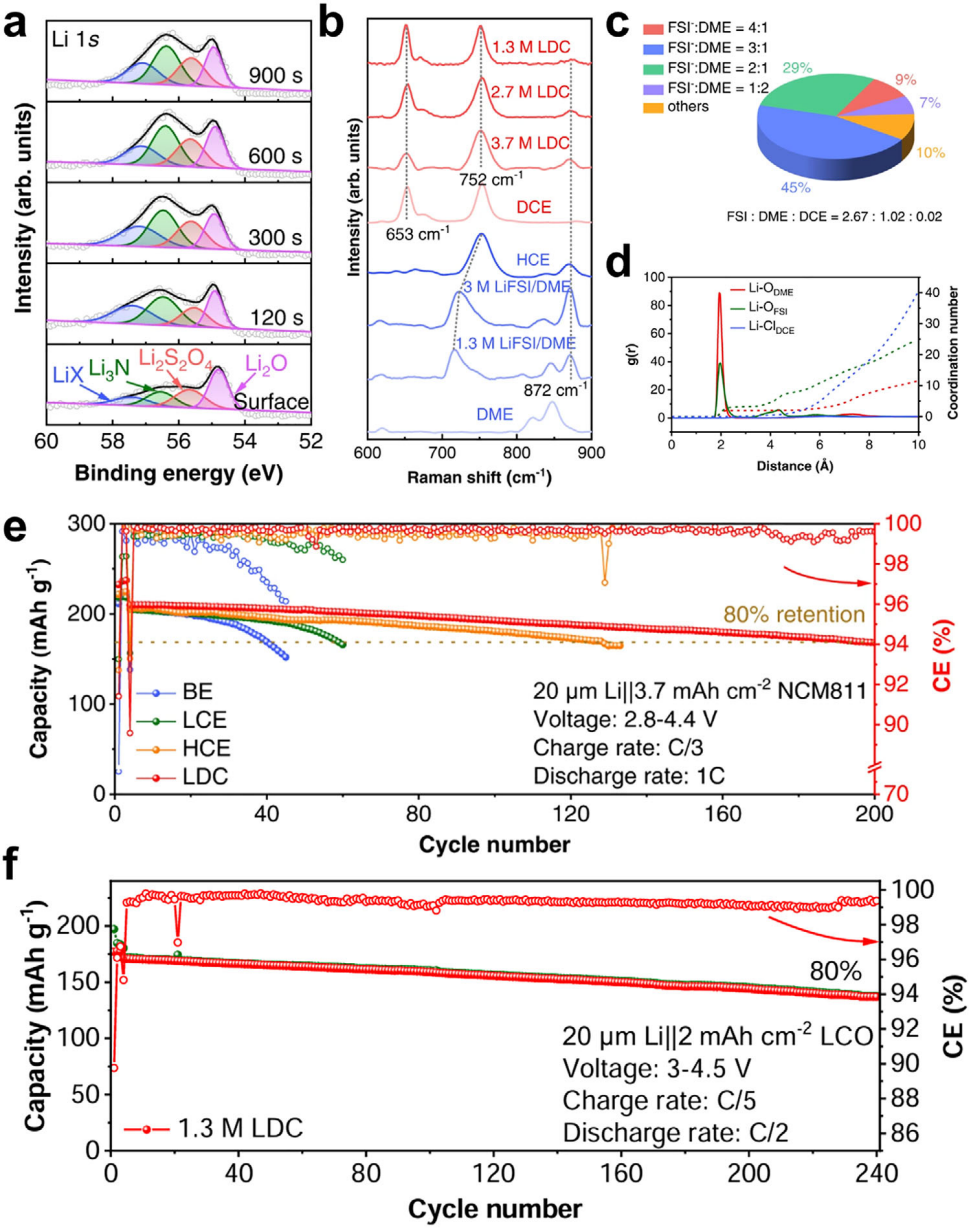
(a) XPS depth profiles of Li 1 s spectra. (b) Raman spectra of the solvents and electrolytes. (c) Proportion of FSI^−^/DME with different ratios in 1.3 m LDC electrolyte. (d) RDF of 1.3 m LDC electrolyte. (e) Cycling performance of Li||NCM811 cells with different electrolytes (1 C = 200 mA g^−1^). (f) Cycling performance of 20 µm Li||2 mAh cm^−2^ LCO cell with 1.3 m LDC. Reproduced with permission [38]. Copyright 2022, Nature Publishing Group.

Additionally, studies have reported that introducing chlorinated diluents can help to form a LiCl‐included SEI layer with robust mechanical properties, thereby effectively suppressing the growth of Li dendrites [38, 66]. For instance, Fan et al., designed a dual‐halide electrolyte (1.3 m LDC) composed of LiFSI, dimethoxyethane (DME), and DCE to form a dual‐halide (LiF_1‐_ *_x_* Cl*_x_*) SEI layer (Figure 5a) [38]. Due to the low ionic migration energy barrier and high surface energy of LiCl, Cl doping endows LiF_1‐_ *_x_* Cl*_x_* with rapid Li^+^ conduction ability while maintaining sufficient mechanical strength. According to the Raman spectroscopy and molecular dynamics (MD) simulations, FSI^−^ in the LDC electrolyte participates in the Li^+^ solvation structure in the form of CIPs or AGGs, and the AGGs solvation structure is dominant (Figure 5b,c). The sharp peak at 2 Å in the RDF corresponds to the close contact of Li^+^/DME and Li^+^/FSI^−^, while the weak peak at 6.5 Å of the Li‐Cl_DCE_ pair suggests a weak interaction between Li^+^ and DCE molecules (Figure 5d). Based on the X‐ray photoelectron spectroscopy (XPS) and MD simulation results, it is inferred that during Li deposition in the LDC electrolyte, FSI^−^ decomposes preferentially, accompanied by weak decomposition of DCE to form LiF_1‐_ *_x_* Cl*_x_*. In the LDC electrolyte, the deposited Li exhibits a dense and dendrite‐free morphology. The cell assembled with LDC electrolyte, LiNi_0.8_Co_0.1_Mn_0.1_O_2_ (NCM811) cathode (≥3.7 mAh cm^−2^) and Li metal anode (20 µm) presents a cycle life of up to 200 cycles (Figure 5e). Meanwhile, the LDC electrolyte is also compatible with the 4.5 V LiCoO_2_ (LCO) cathode, and the corresponding Li||LCO cell (20 µm Li, 2 mAh cm^−2^ LCO) achieves a capacity retention of 80% after 240 cycles (Figure 5f).

**FIGURE 6:**
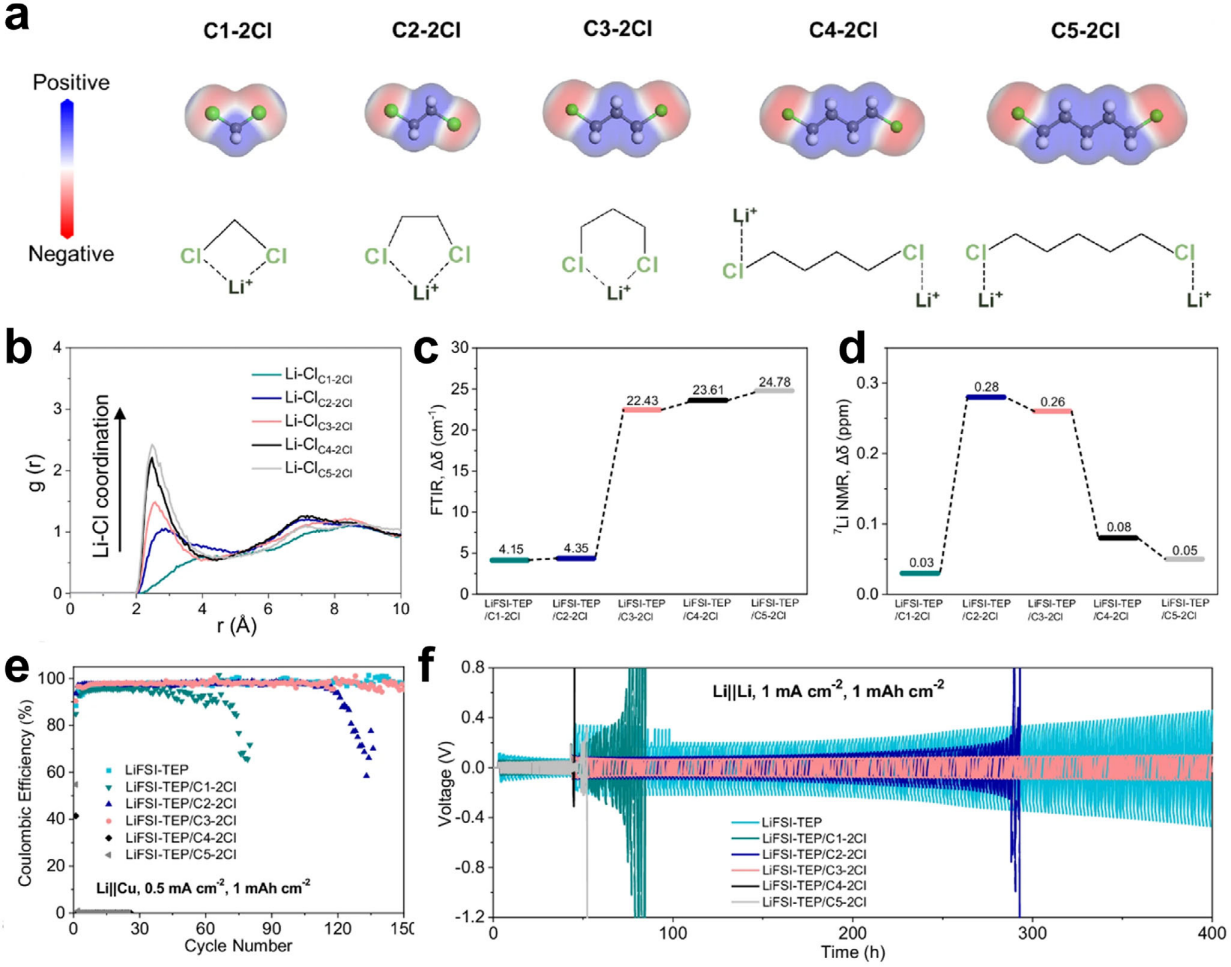
(a) Electrostatic potential of various C‐2Cl diluents and the coordination structures of Li^+^ with different C‐2Cl diluents. Color code: C‐dark gray, H‐white, Cl‐green. (b) RDF curves of Li‐Cl coordination in electrolytes with different C‐2Cl diluents. (c) Δδ of C‐Cl characteristic peaks in different electrolytes obtained by Fourier transform infrared. (d) Δδ of Li^+^ characteristic peaks in different electrolytes obtain by ^7^Li nuclear magnetic resonance (NMR). (e) Cycling performance of Li| |Cu cells in different electrolytes under conditions of 0.5 mA cm^−2^ and 1 mAh cm^−2^. (f) Long‐term Li plating/stripping performance of Li| |Li symmetric cells in different electrolytes. Reproduced with permission [44]. Copyright 2025, Nature Publishing Group.

The influence of chlorinated diluents on the electrolyte performance is not constant. Instead, their roles are governed by how molecular structure regulates Li^+^‐diluent coordination valence, geometry, and electronic stability. Wu et al., systematically elucidated the regulatory mechanisms by which dichloroalkane diluents (C‐2Cl) with different carbon chain lengths influence the solvation structure and electrochemical stability of triethyl phosphate‐based highly concentrated electrolytes through multiscale characterization and theoretical calculations [44]. Analysis of electrostatic potential distributions and molecular dynamics simulations reveals that short‐chain C‐2Cl species with carbon chain lengths of no more than three can form stable cyclic chelation structures with Li^+^, while the Li‐Cl coordination interaction is progressively strengthened with increasing carbon chain length (Figure 6a,b). Infrared spectroscopy and nuclear magnetic resonance results further confirm that C3‐2Cl, namely DCP13, exhibits the most favorable weak coordination characteristics and electronic shielding effect because it forms the most stable six‐membered ring chelation structure, thereby emerging as the optimal diluent (Figure 6c,d). Electrochemical tests demonstrate that only the C3‐2Cl system enables long‐term stable Li plating and stripping, accompanied by low interfacial polarization and negligible side reactions (Figure 6e,f). The optimized C3‐2Cl diluent constructs an anion‐dominated solvation structure, thereby markedly enhancing the cycling stability of LMBs. Additionally, an optimal composition is observed at an intermediate ratio, where weakly coordinating C3‐2Cl reduces the Li^+^ desolvation barrier without disrupting the anion‐dominated solvation structure, enabling the formation of thin and stable inorganic‐rich interphases and improved electrochemical performance. However, when the diluent fraction becomes excessive, the localized high‐concentration structure is weakened, resulting in increased side reactions and degraded performance. This work offers a new strategy for developing low‐cost, safe, and wide‐temperature‐range electrolytes for practical LMBs.

### 3.2 Halogen‐Free Diluents

Halogen‐free diluents can be classified into π‐conjugated aromatics (e.g., benzene, anisole, and ethoxybenzene) and non‐polar alkanes (e.g., *n*‐heptane and *n*‐hexane), each exhibiting distinct solvation motifs and degradation tendencies. Compared with chlorinated diluents, π‐conjugated aromatic diluents exhibit different behavior. Owing to their delocalized electronic structures and lack of strong coordinating functional groups, these molecules show extremely low coordination numbers and weak binding energies with Li^+^, rendering them effectively inert toward the primary solvation sheath. Their role is therefore primarily to reduce viscosity while preserving the intrinsic solvation structure. In addition, their conjugated structures contribute to relatively high oxidative stability. However, their limited participation in interfacial reactions means that the formation of the solid electrolyte interphase is still dominated by anion decomposition. Consequently, their main limitations arise from insufficient interfacial functionality and intrinsic safety concerns, such as low flash points and high flammability.

**FIGURE 7:**
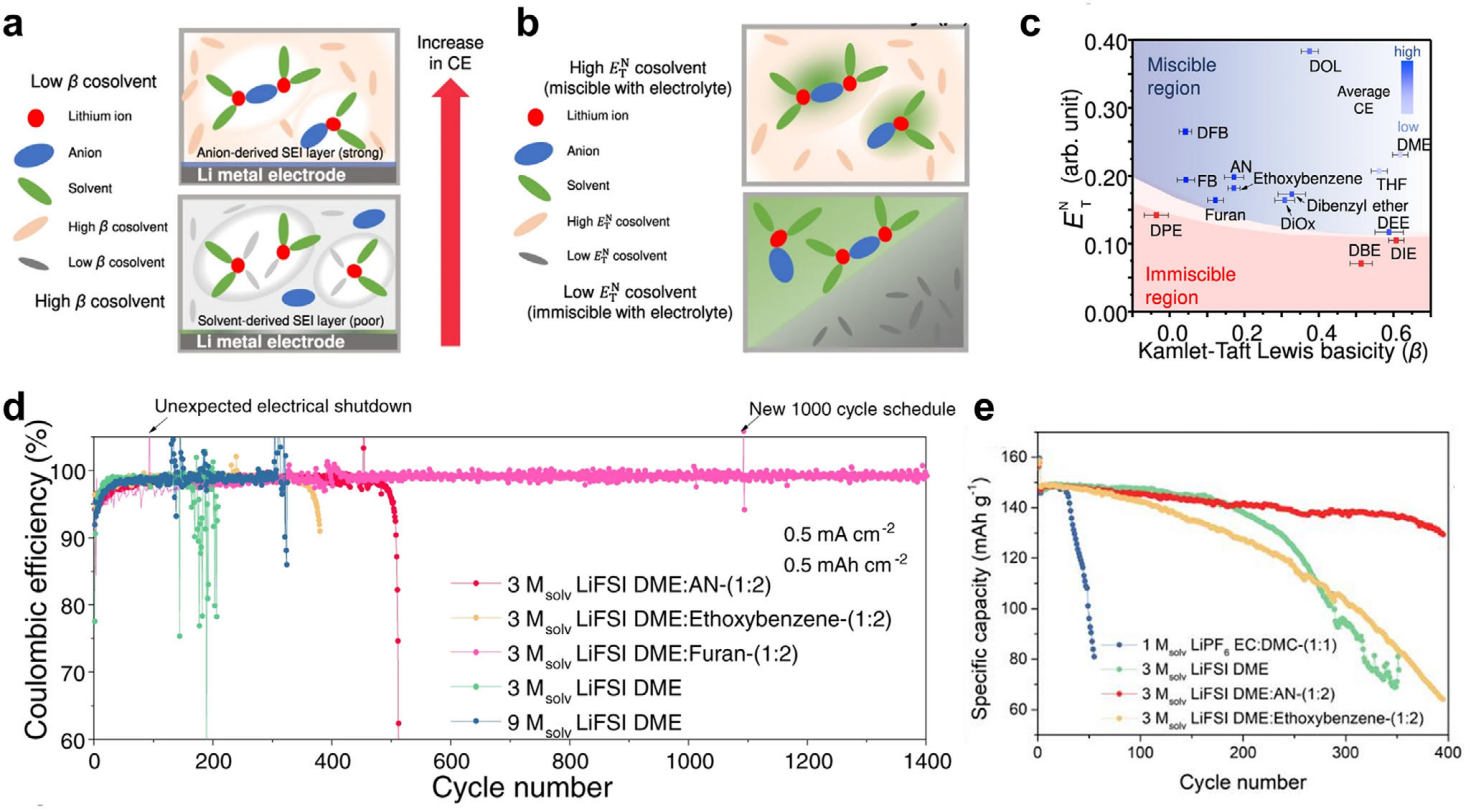
(a) Schematic of the correlation between *β* and the performance (CE) of LMBs employing different electrolytes. (b) Schematic of the correlation between $E_{T}^{N}$ and the miscibility of electrolytes for LMBs. (c) Two‐dimensional plot of the *β* vs. $E_{T}^{N}$ for each solvent. (d) CE of Li|Cu coin cells with different electrolytes at 0.5 mA cm^−2^ and 0.5 mAh cm^−2^. (e) Cycling test of electrochemically deposited thin‐foil Li|LFP full cell. Reproduced with permission [39]. Copyright 2022, Nature Publishing Group.

Lim et al., proposed the design principles and key parameters for the optimal non‐solvating cosolvents of LHCEs and established the correlation between the performance of Li metal anodes and *β* as well as $E_{T}^{N}$ (Figure 7a,b) [39]. Specifically, the optimal parameters are defined as a low *β* value (< 0.2) and medium‐to‐high $E_{T}^{N}$ value (>0.11) (Figure 7c). Furthermore, based on these design principles, non‐fluorinated non‐solvating cosolvents with excellent redox stability (i.e., AN, ethoxybenzene, and furan) were successfully screened. The results show that the performance of the cells using AN, ethoxybenzene, or furan as diluents was superior to that of the DME‐based cell without any diluent. Specifically, the cell using AN achieves an average CE of 98.5% after 500 cycles, while the one using furan maintains an average CE of 99.0% after 1400 cycles (Figure 7d). Full‐cell cycling tests were conducted using LiFePO_4_ (LFP) as the cathode and thin electrodeposited Li metal foil as the anode. The capacity retention of the cell without diluent (3 M_solv_ LiFSI DME) after 300 cycles is only 63.2%, while the capacity retentions of the cells with AN (3 M_solv_ LiFSI DME: AN‐(1:2)) and ethoxybenzene (3 M_solv_ LiFSI DME:ethoxybenzene‐(1:2)) after 300 cycles are 93.7% and 71.4%, respectively (Figure 7e). Of note, for electrolytes using DME as the main solvent and AN or furan as diluents, increasing the diluent fraction promotes anion participation in the solvation structure and facilitates the formation of dense interphases. However, excessive dilution reduces miscibility and ionic conductivity. As reported, an optimal electrochemical performance is achieved at a DME to diluent volume ratio of 1:2, whereas further increasing the diluent content leads to deteriorated transport properties and compromised cycling stability. This work is of great significance for promoting the design of high‐performance electrolytes for LMBs.

**FIGURE 8:**
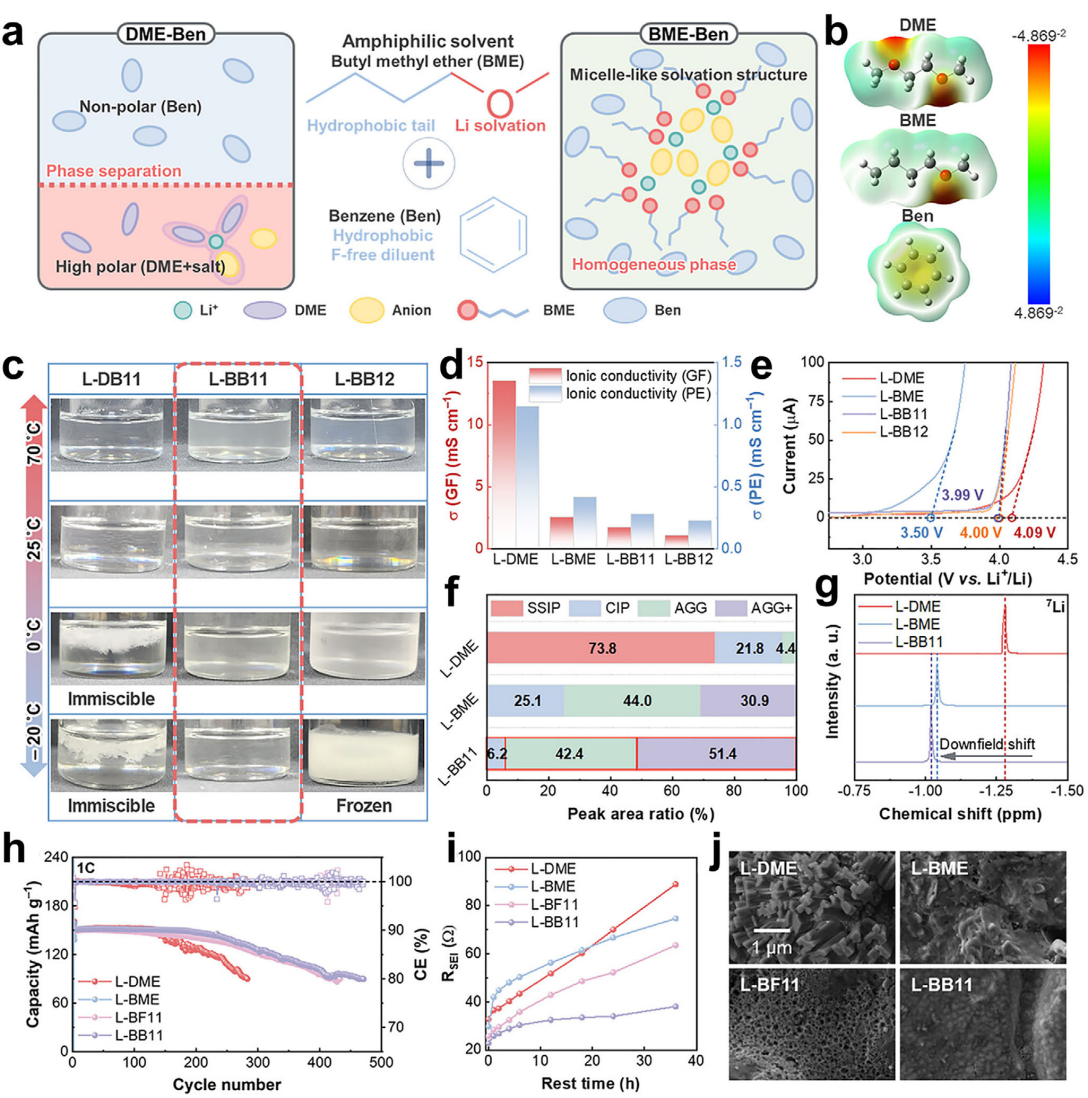
(a) Schematic representation of DME‐benzene and BME‐benzene systems highlighting miscibility. (b) Molecular electrostatic potential (MEP) maps of individual solvents and diluents; scale bars in MEP maps represent the magnitude of electrostatic potential. (c) Miscibility tests of DME‐benzene and BME‐benzene systems. Electrochemical properties including (d) ionic conductivity, (e) oxidative stability, (f) relative populations of FSI^−^ anion states, and (g) ^7^Li NMR chemical shifts. (h) Cycling performance of Li||LFP full cells. (i) Time‐dependent changes in SEI resistance during rest periods. (j) SEM images of Li metal after 72 h immersion in each electrolyte. Reproduced with permission [45]. Copyright 2026, Wiley‐VCH GmbH.

Lim et al., developed a non‐fluorinated LHCE by employing benzene as a novel diluent in combination with amphiphilic butyl methyl ether (BME) as a co‐solvent, thereby addressing the issues associated with conventional fluorinated diluents, including Li metal corrosion, high toxicity, and elevated cost [45]. The commonly used strongly polar solvent DME is immiscible with nonpolar benzene due to polarity mismatch, whereas BME, which contains both polar ether oxygen and a nonpolar alkyl chain, serves as a molecular bridge that enables homogeneous compatibility between benzene and the electrolyte (Figure 8a). The molecular electrostatic potential distribution further confirms this amphiphilic characteristic (Figure 8b). Compared with L‐DB11 with a DME to benzene ratio of 1:1, and L‐BB12 with a BME to benzene ratio of 1:2, L‐BB11 with a BME to benzene ratio of 1:1 maintains a homogeneous and stable phase without separation even at low temperature (Figure 8c). The ionic conductivity of the electrolyte system gradually decreases with increasing benzene content (Figure 8d). While L‐BB11 still satisfies practical requirements, L‐BB12 exhibits a further reduction. The oxidation stability of L‐BME without benzene is approximately 3.5 V, which increases to about 4.0 V for both L‐BB11 and L‐BB12 upon the introduction of benzene (Figure 8e). Raman and NMR analyses reveal that the L‐BB11 system forms an anion‐dominated solvation structure primarily consisting of AGG and AGG^+^, effectively suppressing side reactions associated with free solvent molecules (Figure 8f,g). Electrochemical and interfacial characterization results demonstrate that L‐BB11 enables long‐term stable cycling in Li||LFP full cells. During resting, no significant increase in solid electrolyte interphase resistance is observed, and the Li metal surface remains free of noticeable corrosion, maintaining a dense and smooth morphology (Figure 8h–j). Overall, this work provides a feasible new strategy for developing low‐cost, environmentally benign, and highly stable electrolytes for LMBs.

Non‐polar alkanes can be regarded as nearly ideal non‐solvating diluents from a thermodynamic perspective. Their extremely low polarity results in negligible interaction with Li^+^, ensuring that they do not interfere with the local high‐concentration solvation structure. Their primary function is therefore physical dilution, effectively reducing viscosity while maintaining the integrity of the electrolyte microstructure. However, their lack of polarity also limits their ability to support ion transport and interfacial stabilization. Notably, compared with chlorinated solvents and aromatic hydrocarbons, linear alkanes exhibit improved biodegradability and eliminate halogen‐related environmental concerns, rendering them relatively closer to “green” solvents in a comparative sense. Nevertheless, their extreme flammability and volatility impose intrinsic safety limitations that preclude their classification as intrinsically sustainable electrolyte components. In this regard, linear alkanes should be viewed as transitional, non‐fluorinated diluents that provide valuable mechanistic benchmarks for LHCEs design. Future efforts should therefore focus on the rational development of non‐fluorinated diluents that retain weak solvation characteristics while simultaneously achieving reduced flammability, benign toxicological profiles, and regulatory compatibility, thereby advancing toward truly sustainable electrolyte systems.

As presented in Table 2, various advanced LHCEs with non‐fluorinated diluents for LMBs are summarized, showing their respective electrochemical properties. It should be noted that the electrochemical performance data of different electrolyte systems were obtained under distinct test conditions. The table is intended to illustrate the diversity of reported performance rather than to establish direct equivalency across systems. Additionally, we call for the gradual establishment of more unified and standardized testing conditions and data reporting protocols. It is recommended that practically relevant testing conditions be adopted to better reflect real‐world application scenarios. Such standardization would not only facilitate meaningful cross‐study comparisons but also accelerate the rational design and optimization of non‐fluorinated diluents for high‐performance and sustainable LMBs.

**TABLE 2:** Electrochemical properties of LHCEs with different non‐fluorinated diluents.

Electrolyte formula	Li‐Cu cell condition	Coulombic efficiency@cycle numbers	Li‐Li symmetric cell condition	Cycle time	Cell configuration	Capacity retention @cycle number/cycle condition	Refs.
**Chlorinated Diluents**							
1.3 m LiFSI/DME‐DCE (0.949:1:6, by mol.)	0.5 mA cm^−^^2^, 1 mAh cm^−^^2^	99.54%@40cycles	0.5 mA cm^−^^2^, 1 mAh cm^−^^2^	1500 h	20 µm Li||3.7 mAh cm^−^^2^ NCM811 (4.4 V)	80%@200cycles/ 0.33 C, 1 C	[38]
					20 µm Li||2.0 mAh cm^−^^2^ LCO (4.5 V)	80%240cycles/ 0.2 C, 0.5 C	
1 m (mol kg^−1^) LiFSI/Pyr_13_FSI‐DCP13 (2:3, by mol.)	1 mA cm^−^^2^, 0.5 mAh cm^−^^2^	99.1%@900cycles	1 mA cm^−^^2^, 2 mAh cm^−^^2^	1500 h	450 µm Li||13.1 mg cm^−^^2^ NCM90 (4.3 V, RT)	94%@240cycles/ 0.3 C, 0.3 C	[43]
					450 µm Li||13.1 mg cm^−^^2^ NCM90 (4.4 V, RT)	95.5%@100cycles/ 0.3 C, 0.3 C	
					450 µm Li||13.1 mg cm^−^^2^ NCM90 (4.5 V, RT)	99.5%@100cycles/ 0.3 C, 0.3 C	
					450 µm Li||13.1 mg cm^−^^2^ NCM90 (4.6 V, RT)	88.5%@100cycles/ 0.3 C, 0.3 C	
					450 µm Li||13.1 mg cm^−^^2^ NCM90 (4.4 V, 60 °C)	85%@70cycles/ 0.3 C, 0.3 C	
					450 µm Li||13.1 mg cm^−^^2^ NCM90 (4.4 V, −20 °C)	88%@70cycles/ 0.1 C, 0.1 C	
LiFSI‐TEP/C3‐2Cl (1:1.5:3, by mol.)	0.5 mA cm^−^^2^ 1 mAh cm^−^^2^	98%@60cycles	1 mA cm^−^^2,^ 1 mAh cm^−^^2^	400 h	50 µm Li||2.0 mAh cm^−^^2^ NCM523 (4.5 V)	81.7%@200cycles/ 0.5 C, 0.5 C	[44]
0.68 m LiFSI‐C3mpyrFSI/DCM (1:1.3, by mol.)	1 mA cm^−^^2^, 0.5 mAh cm^−^^2^	99.2%@550cycles	1 mA cm^−^^2^, 1 mAh cm^−^^2^	900 h	Li||11.5 mg cm^−^^2^ LCO (4.3 V)	78.9%@150cycles/ 0.3 C, 0.3 C	[66]
					Li||11.5 mg cm^−^^2^ LCO (4.4 V)	98.9%@100cycles/ 0.3 C, 0.3 C	
					Li||11.5 mg cm^−^^2^ LCO (4.5 V)	98.8%@100cycles/ 0.3 C, 0.3 C	
					50 µm Li||11.5 mg cm^−^^2^ LCO (4.4 V)	80.6%@70cycles/ 0.3 C, 0.3 C	
					Li||11.5 mg cm^−^^2^ LCO (4.3 V, 60°C)	98.1%@100cycles/ 0.5 C, 0.5 C	
					Li||11.5 mg/cm^2^ LCO (4.3 V, ‐20°C)	87.7%@20cycles/ 0.05 C, 0.05 C	
2 m LiFSI DME/CIDEE (1:7 by vol.)	0.5 mA cm^−^^2^, 1 mAh cm^−^^2^	99.48%@500cycles	1 mA cm^−^^2^, 3 mAh cm^−^^2^	2000 h	20 µm Li||2.5 mAh cm^−^^2^ NCM811 (4.4 V)	80%@429cycles/ 0.3 C, 0.3 C	[70]
					20 µm Li||3.8 mAh cm^−^^2^ NCM811 (4.3 V)	80%@350cycles/ 0.2 C, 0.2 C	
			0.5 mA cm^−^^2^, 2 mAh cm^−^^2^	3200 h	100 µm Li||30 mg cm^−^^2^ NCM811	84%@116cycles/ 0.1 C, 0.25 C	
					Cu||3.8 mAh cm^−^^2^ NCM811 (4.2 V)	70%@90cycles/ 0.2 C, 0.5 C	
10 m LiFSI in DME	0.5 mA cm^−^^2^, 1 mAh cm^−^^2^	99.28%@500cycles	/	/	20 µm Li||2.5 mAh cm^−^^2^ NCM811 (4.4 V)	80%@300cycles/ 0.3 C, 0.3C	
**Halogen‐Free Diluents**							
3 M_solv_ LiFSI DME/AN (1:2, by vol.)	0.5 mA cm^−^^2^, 0.5 mAh cm^−^^2^	98.5%@500cycles	/	/	300 µm Li||11 mg cm^−^^2^ LFP	93.7%@300cycles/ 0.5 C, 0.5 C	[39]
					Li ||21 mg cm^−^^2^ LFP (N/P ratio∼1.6)	86.8%@125cycles/ 0.2 C, 0.2 C	
3M_solv_ LiFSI DME/Ethoxybenzene (1:2, by vol.)	/	/	/	/	300 µm Li||11 mg cm^−^^2^ LFP	71.4%@300cycles/ 0.5 C, 0.5 C	
					Li ||21 mg cm^−^^2^ LFP (N/P ratio∼1.6)	83.4%@125cycles/ 0.2 C, 0.2 C	
1 M_solv_ LiFSI DME/Furan (1:2, by vol.)	/	/	/	/	300 µm Li||11 mg cm^−^^2^ LFP	94.8%@300cycles/ 0.5 C, 0.5 C	
					Li||21 mg cm^−^^2^ LFP (N/P ratio∼1.6)	94.6%@125cycles/ 0.2 C, 0.2 C	
2 M_solv_ LiFSI DME/Furan (1:2, by vol.)	/	/	/	/	300 µm Li||11 mg cm^−^^2^ LFP	86.2%@300cycles/ 0.5 C, 0.5 C	
					Li||21 mg cm^−^^2^ LFP (N/P ratio∼1.6)	94%@125cycles/ 0.2 C, 0.2 C	
3 M_solv_ LiFSI DME/Furan (1:2, by vol.)	0.5 mA cm^−^^2^, 0.5 mAh cm^−^^2^	99.0%@1400cycles	/	/	/	/	
	2 mA cm^−^^2^, 1 mAh cm^−^^2^	99.4%@500cycles					
1.6 m LiFSI+0.4 M LiTFSI HME/HEP (1:1, by vol.)	0.5 mA cm^−^^2^, 1 mAh cm^−^^2^	99.16%@30‐100cycles, 99.29%@100‐200cycles	/	/	Li||1 mg cm^−^^2^ PTCDA (3.0 V, 25°C)	98.2%@200cycles/ 1 C, 1 C	[40]
					Li||1 mg cm^−^^2^ PTCDA (3.0 V, 60°C)	81%@105cycles/ 1 C, 1 C	
2 m LiFSI‐DEE/PhH (1:1, by vol.)	1 mA cm^2^, 1 mAh cm^−^^2^	99.4%@500cycles	1 mA cm^−^^2^, 1 mAh cm^−^^2^	900 h	Li||9 mg cm^−^^2^ SC811 (4.3 V)	87.3%@450cycles/ 0.5 C, 0.5 C	[41]
					50 µm Li||31 mg cm^−^^2^ Ni83 (4.3 V)	87%@70cycles/ 1.2 mA cm^−^^2^ charge, 2.4 mA cm^−^^2^ discharge	
1.5 m LiFSI, BME/Benzene (1:1, by mol.)	1 mA cm^−^^2^, 1 mAh cm^−^^2^	99.43%@200cycles	1 mA cm^−^^2^, 1 mAh cm^−^^2^	1500 h	20 µm Li||9.5 mg cm^−^^2^ LFP (4.0 V)	50%@∼450cycles/ 1 C, 1 C	[45]
			3 mA cm^−^^2^, 1 mAh cm^−^^2^	400 h			
LiFSI:EmimFSI:Anisole (1:2:6, by mol.)	stripping at 1.5 mA cm^−^^2^, plating at 0.5 mA cm^−^^2^	99.71%@50cycles	1 mA cm^−^^2^, 1 mAh cm^−^^2^	900 h	500 µm Li||10 mg cm^−^^2^ LFP (3.6 V)	No fading@400cycles/ 0.33 C, 1 C	[68]
					2.25 mAh cm^−^^2^ Li||10 mg cm^−^^2^ LFP (3.6 V)	94%@400cycles/ 0.33 C, 1 C	
					3.75 mAh cm^−^^2^ Li||2.8 mg cm^−^^2^ SPAN (3.0 V)	90%@350cycles/ 0.33 C, 1 C	

M_solv_ denotes the molality of Li salt normalized to the mass of coordinating solvent, rather than the total electrolyte mass. This definition is adopted to maintain consistency with the referenced literature and to more accurately describe the salt‐solvent ratio in LHCE systems.

## 4 Summary and Outlook

In summary, non‐fluorinated diluents have emerged as a promising pathway for constructing LHCEs, yet their behavior deviates substantially from simplified expectations. As discussed throughout this perspective, non‐fluorinated diluents are not universally non‐solvating, intrinsically safe, or inherently sustainable. Instead, their physicochemical roles are governed by a delicate interplay between weak coordination, non‐equilibrium solvation participation, and interfacial reactivity, which collectively determine electrolyte performance. At the same time, practical constraints, including compositional boundaries, flammability, volatility, and potential toxicity, impose additional limitations on their real‐world applicability. These observations highlight that the evaluation of non‐fluorinated diluents must extend beyond electrochemical metrics to incorporate safety, scalability, and regulatory considerations. Looking forward, the development of non‐fluorinated diluents should be guided by several prioritized and testable research directions.

First, targeted design of non‐fluorinated diluents can be achieved by leveraging DFT and MD simulations. In essence, the core of performance optimization for non‐fluorinated diluents lies in the precise matching of “structure‐parameter‐function”. Key parameters such as dielectric constant, binding energy, CN, and electrochemical window of molecules are predicted to precisely control the alkyl chain length, branched chain structure, and functional group, thereby leading to the development of new non‐fluorinated diluents with low viscosity, high stability, and wide miscibility range.

Second, constructing multi‐component composite systems is an effective approach to overcome the performance limitations of a single non‐fluorinated diluent. The non‐fluorinated/fluorinated composite diluents can serve as a short‐term transitional solution [42]. In the long term, the construction of a fully non‐fluorinated electrolyte system will be the ultimate goal [2, 71, 72]. In addition, the development of non‐fluorinated binders may further support the transition toward fully fluorine‐free battery systems from a holistic and sustainable perspective.

Third, the complexity of interface reactions requires the adoption of advanced in situ characterization techniques for dynamic, real‐time, and quantitative analysis. At the current stage, in situ experimental techniques alone are not able to directly quantify the ionic conductivity of an individual SEI layer. One possible approach is to combine in situ characterization techniques with electrochemical impedance spectroscopy, together with advanced modeling and simulation methods such as multiphysics modeling or atomistic simulations, to gain deeper insight into the respective contributions of different SEI components. Although this strategy remains challenging, it holds promise for enabling the semi‐quantitative estimation of the ionic conductivity of an individual SEI layer under appropriate conditions. Importantly, to establish the causal relationship between the solvation structure and interface properties, future research needs to design key control experiments to verify the proposed mechanism, such as isotope labeling and multi‐scale characterization techniques.

Fourth, advancing the industrialization of non‐fluorinated diluents requires simultaneous progress in cost‐effective synthesis, process compatibility, and safety‐aware engineering design. For chemically synthesized non‐fluorinated diluents, optimization of catalyst systems and reaction pathways is essential to enable scalable and continuous production. In addition to manufacturing scalability, intrinsic safety‐related constraints of certain non‐fluorinated diluents must be explicitly considered at the industrial level. In particular, alkane‐based diluents (e.g., *n*‐hexane and *n*‐heptane), while effective in constructing weakly solvating and anion‐rich solvation environments, inherently exhibit low flash points and high volatility, which may impose practical limitations during electrolyte handling and cell fabrication. To mitigate these risks, future efforts should integrate formulation‐level strategies, such as flame‐retardant main solvent systems or gel‐based electrolytes to suppress volatility, together with engineering controls, including sealed cell architectures that reduce flammability hazards under realistic operating conditions. Finally, efforts should be directed toward establishing standardized full life cycle assessment frameworks encompassing raw material acquisition, synthesis, application, and end‐of‐life recycling. Within this framework, the practical advantages of non‐fluorinated diluents in terms of environmental impact, economic feasibility, safety compliance, and carbon footprint should be quantitatively evaluated, ensuring that their industrial deployment is guided by holistic and application‐relevant sustainability metrics rather than single‐parameter performance advantages.

Future efforts should therefore focus on the rational development of non‐fluorinated diluents that retain weak solvation characteristics while simultaneously achieving reduced flammability, benign toxicological profiles, and regulatory compatibility, thereby advancing toward truly sustainable electrolyte systems. With the advancement of molecular design for non‐fluorinated diluents and the development of battery technologies, the synergistic integration of non‐fluorinated electrolytes with high‐capacity cathode materials (e.g., Li‐rich manganese‐based oxides, sulfur, and oxygen) can be realized in the future, facilitating the practical application of ultra‐high energy density LMBs. The current research on non‐fluorinated diluents‐based LHCEs can be further expanded to other alkali metal systems, such as sodium metal batteries and potassium metal batteries [73]. As the core carrier for more sustainable electrolytes, non‐fluorinated diluents will play a pivotal role in applications such as electric vehicles and large‐scale energy storage, providing high‐performance and environmentally friendly energy storage technology guarantees for the global energy transition.

## Conflicts of Interest

The authors declare no conflicts of interest.

## Data Availability

Data availability is not applicable to this article as no new data were created or analyzed in this study.
